# FROM NATIONAL DATA TO LOCAL APPLICATION: INNOVATIONS IN AGING NETWORK SERVICES AND RESEARCH PARTNERSHIPS

**DOI:** 10.1093/geroni/igae098.0644

**Published:** 2024-12-31

**Authors:** Traci Wilson, Marisa Sheldon, Heather Menne

**Affiliations:** Chair; Co-Chair; Discussant

## Abstract

As the local experts in planning, coordinating, and implementing aging services, Area Agencies on Aging (AAAs) address a broad range of social and health needs of an increasingly diverse older adult population. To live independently and with optimal health in their homes and communities, many older adults depend on Older Americans Act (OAA)-funded services such as nutrition and in-home supportive services, which they access through local AAAs. Increasingly, older adults look to AAAs for supports outside of core OAA services, such as housing and emergency preparedness and response. This symposium will provide data-driven insights on adaptive strategies to enhance aging services both within and beyond the OAA and how AAAs and researchers can collaborate to support innovations. Each presenter represents a different level and perspective within the Aging Network. The first speaker from USAging will use the National Survey of AAAs to describe organizational characteristics that support aging services innovations. The presenter from the Administration for Community Living will describe findings from recent National Surveys of OAA Participants including how they adapted during COVID and how prepared they are for future emergencies. The director of a university research center will demonstrate the continuum of community engaged research approaches, through a bi-directional relationship with an AAA. The symposium will conclude with a presentation from an AAA illustrating how her agency engages with a university-based research center to innovate, build evidence for program effectiveness, and strengthen services for at-risk older adults.

